# A pathway to greater meaning in life and well-being for senior executives beset by anti-meaning

**DOI:** 10.3389/fpsyg.2023.1187913

**Published:** 2023-07-18

**Authors:** Juan-Mari Kruger, Jeremias Jesaja De Klerk

**Affiliations:** Stellenbosch Business School, Stellenbosch University, Stellenbosch, South Africa

**Keywords:** life meaning, meaning in life, anti-meaning, senior executive, well-being, wellness, work–life balance

## Abstract

**Introduction:**

Although work is a significant source of meaning for most people, the role of senior executive generates different meaning and well-being complexities than those experienced or faced by general employees. This study explored how meaning and anti-meaning components affect senior executives’ experiences of meaning in life and well-being. The findings enabled devising a pathway to enhance senior executives’ net experiences of meaning in life and well-being.

**Methods:**

A cross-sectional, semi-structured interview study design was used to gather rich qualitative data. Eight participants from southern and eastern Africa, who had held the position of chief executive officer or managing director for at least five years, were interviewed.

**Results:**

The findings demonstrated that senior executives’ work roles provide a significant source of meaning. However, the roles are accompanied by unavoidable anti-meanings, which are likely to generate additional anti-meanings if not tempered sufficiently, thus reducing the net meaning experienced.

**Discussion:**

From the findings, a practical pathway was devised to assist top executives to deal with the bipolar relationship between meaning and anti-meaning. Consulting and counseling practitioners can utilize the pathway to guide, support, and counsel senior executives towards improved meaning, temper anti-meaning and improve well-being.

## 1. Introduction

Meaning in life refers to the subjective experiences of one’s life as meaningful and significant in some way (Steger, 2018; Li et al., 2021). According to Frankl (1984), the primary motivational force of humans is a will or desire to find meaning in their lives. Meaning in life refers to subjectively experiencing one’s life as being meaningful in some way (Steger, 2018; Li et al., 2021). Meaning in life implies a feeling that one’s life matters and makes sense, and a conviction of a higher purpose for one’s life (De Klerk, 2023). Research has consistently confirmed meaning in life correlates with general well-being, psychological health, and even physical health (Steger, 2018; Arslan and Allen, 2022). Indeed, a sense of meaning in life appears to be a critical element of healthy functioning (De Klerk, 2005; Steger, 2012; Salicru, 2021). Although the concept of meaning in life was recognized as prominent in the fields of existential philosophy, psychiatry and clinical psychology (Vos and Vitali, 2018), it was also shown to be imperative in the world of work (De Klerk, 2005, 2023; Steger, 2019). As an important domain of life, work is a significant source of meaning (De Klerk et al., 2006), and many people find meaning through their work (Steger, 2019; De Klerk, 2023). The sense of meaning that individuals often derive from their work has been well documented (Steger, 2017, 2019; De Klerk, 2023). However, the role of senior executive generates different meaning and well-being complexities than those experienced or faced by general employees.

The construct of meaning in life was originally conceptualized as a monopolar construct on a continuum from meaningful to meaningless (Metz, 2013; Nyholm and Campbell, 2022). However, even meaningful activities can reduce an executive’s net experience of meaning when accompanied by unavoidable side effects that are often experienced by top executives, such as excessive stress (Rook et al., 2016) and loneliness (Siqueira et al., 2019). The monopolar view of meaning ultimately fails to account adequately for activities that are not merely meaningless, but directly oppose and subtract from the overall meaning experience (Nyholm, 2021; Nyholm and Campbell, 2022). Whereas meaninglessness represents the absence of meaning (De Klerk et al., 2009), Nyholm and Campbell (2022) defined anti-meaning as those aspects that subtract from an individual’s experience of meaning. Meaning and anti-meaning can thus be regarded as antagonistic forces, whose net effect determines the final level of meaningfulness that an individual experiences (Campbell and Nyholm, 2015).

There is limited research available that focused specifically on senior executives’ experience of meaning in life and how it relates to their well-being (Jones, 2019). As far as we could establish, there is no research available on the extent to which senior executives perceive and experience anti-meaning. Faced with significant pressure, senior executives need an alternative model to maintain their overall sense of meaning to improve their well-being. Viewing meaning as a bipolar construct allowed us to gain a more holistic understanding of executives’ net experience of meaning. This study aims to explore how anti-meaning can be tempered, to enhance senior executives’ net experience of meaning in life and, therefore, also their well-being. The findings provided the foundation to devise a pathway to assist senior executives with a novel approach to optimize the relationship between meaning and anti-meaning. Consulting and counseling practitioners can use the proposed pathway to guide senior executives towards tempering anti-meaning in order to enjoy an improved net experience of meaning and well-being.

## 2. Literature review

### 2.1. Meaning in life and work

According to Frankl (1984), every person has a compelling motivation to find meaning in their lives. Meaning in life is about the extent to which an individual subjectively experiences their life as being meaningful and significant in some way (Steger, 2018; Li et al., 2021; De Klerk, 2023), and as having direction (Martela and Steger, 2016). A sense of meaning in life promotes the conviction that one’s life matters, makes sense, holds positive value, and serves some higher purpose (De Klerk, 2023).

A sense of meaning in life has been shown to originate from a variety of sources, with work often being a prominent source of meaning (Steger, 2012, 2017; Allan et al., 2018; Bailey et al., 2019; De Klerk, 2023). Meaning can also develop from appreciating love, beauty, goodness and nature; experiencing the kindness of others (Worth and Smith, 2021); enjoying meaningful relationships (Hicks et al., 2010; Lambert et al., 2013); and having a sense of social belonging (Lambert et al., 2013; Baumeister and Landau, 2018). Individuals can find meaning and purpose regardless of adverse conditions such as ill health, trauma and chronic stress (Frankl, 1984; Vos and Vitali, 2018). Indeed, meaning can develop from facing adverse circumstances with dignity, courage and virtue (Frankl, 1992; Wong, 2016).

With work being so central to individuals’ existence, their lives often become meaningful through their work and careers (Pignault and Houssemand, 2021). However, this was not necessarily always the case. The ancient Greeks regarded work as a curse reserved for the slaves and the poor (Fayard, 2021). Under the influence of religious indoctrination, attitudes toward work gradually changed and work was seen as good for moral and spiritual integrity (Gill, 1999). Since the Reformation, hard work became a religious obligation and a virtue (i.e., the protestant work ethic) (Thomas, 1999). During the industrial revolution, work was regarded mainly as a means to earn a living. However, this view changed quickly as many of the psychosocial needs that were historically met through social structures and rituals in previous societies have been replaced by the social convention of paid work (Gill, 1999). Indeed, work became one of the most central domains in people’s lives in the modern world (MOW International Research Team, 1987; Allan et al., 2015; Steger, 2019). This development gave rise to studies on job involvement (the psychological identification with one’s job) (MOW International Research Team, 1987; Paullay et al., 1994) and work centrality or work involvement (the degree of importance that work in general plays in a person’s live) (Kanungo, 1982). Work centrality and job involvement typically measure high for senior executives (Judge et al., 1995).

### 2.2. Meaning and well-being

Well-being is a comprehensive and multidimensional construct regarding the physiological, psychological and social facets of people’s lives that produce experiences of “harmony, wellness and balance” (Miller and Foster, 2010, p. 5). Of the many well-being dimensions identified (Miller and Foster, 2010; Quick et al., 2014), psychological (and emotional) well-being, physiological wellness, social wellness, spiritual well-being and work wellness hold particular importance to senior executives and their relationships, and their sense of meaning (Steger, 2018). Psychological wellness entails an individual’s attitudes and beliefs about their life and their mental health (Miller and Foster, 2010). Social wellness involves the depth and breadth of an individual’s interactions and relationships with others, the community, and nature (Bailey and Fernando, 2012; Howell et al., 2013). Interpersonal relationships within one’s immediate social circle, such as with one’s family and friends, enhance one’s sense of belonging, social connectedness, and expected and perceived emotional support (Hicks et al., 2010, 2012; Lambert et al., 2013). Research consistently confirmed a sense of meaning in life to correlate with general well-being, psychological health, social well-being, work wellness, and even physiological health. Table 1 indicates some well-being variables that are correlated with a sense of meaning.

**TABLE 1:** Well-being variables that have been correlated with meaning in life.

Well-being variables	References
**Psychological, social, and work well-being**	
Positive self-concept, self-esteem, and self-worth	Stillman et al., 2011; Vanhooren et al., 2016
Positive lifestyle behaviors	Steptoe and Fancourt, 2019
Improved mental wellness and general well-being	Li et al., 2021
Healthy social functioning, relationships, and social engagement	Stillman et al., 2011; Yu and Chang, 2021
Higher levels of life quality and satisfaction, hope, joy, and love	Brassai et al., 2011; O’Donnell et al., 2014; Tak et al., 2017; Li et al., 2021
Increased resilience, ability to cope with life-stressors	Southwick et al., 2016; Park and Baumeister, 2017; Vos and Vitali, 2018; Weber et al., 2020
Reduced depression and suicidal ideation	Steger et al., 2009; Salicru, 2021
Lower chronic parenting stress	Crosswell et al., 2022
Lower levels of negative emotions	Park and Baumeister, 2017
Reduced psychological distress and mental illness	Shelton et al., 2020
Reduced levels of death anxiety	Dursun et al., 2022
Intrinsic motivation, work commitment, and goal orientation	De Klerk et al., 2006
Job satisfaction	Van der Walt and De Klerk, 2014
**Physiological well-being**	
Higher self-rated health scores	Steptoe and Fancourt, 2019
Reduced unhealthy behaviors, e.g. smoking, drinking, and substance abuse	Brassai et al., 2011; Schnetzer et al., 2013; Steptoe and Fancourt, 2019
Reduction in chronic disease	Steptoe and Fancourt, 2019
Reduced stress-related hormones	Pulopulos and Kozusznik, 2018; Chiang et al., 2019
Improved physical activity and fitness	Steptoe and Fancourt, 2019; Rush et al., 2021
Increased longevity	Trudel-Fitzgerald et al., 2021
Lower levels of obesity and insulin resistance	Steptoe and Fancourt, 2019
Better sleep quality	Steptoe and Fancourt, 2019
Cardiovascular fitness and reduced cardiovascular risk	Quick et al., 2014; Vos, 2021

Work-wellness refers to gaining satisfaction and enrichment from one’s work and the ability to express one’s values at work (Miller and Foster, 2010). The social milieu of the workplace sets the stage for satisfying meaning-related needs, such as connectedness and recognition (Siqueira et al., 2019) and achieving personal goals (De Klerk et al., 2006; Miller and Foster, 2010). Although meaningful work or finding meaning through work does not equate to meaning in life, it is a primary source of personal enrichment and meaning (De Klerk, 2023). Finding meaning in work has been shown to increase engagement, productivity and job satisfaction (Akgunduz et al., 2018; Allan et al., 2019), and enhances individuals’ sense of purpose, efficacy, value and self-worth (Baumeister and Stillman, 2009; Lambert et al., 2012). Meaningful work promotes overall well-being and life satisfaction, while decreasing anxiety, depression and stress (Allan et al., 2019). In contrast, work that is experienced as meaningless has been linked to aspects such as boredom (Hu and Hirsh, 2017) and burnout (Dal Corso et al., 2020). Although work–life balance is generally regarded as an important part of work-wellness (Miller and Foster, 2010; Bloom, 2016; Kelliher et al., 2019), Groysberg and Abrahams (2014) argued that it is an unrealistic goal for executives to achieve the conventional perspective of work–life balance.

Spirituality is about a sense of interconnectedness with the universe, being part of something that transcends the self (Weeks and Schaffert, 2019), and a sense of purpose and meaning (Quick et al., 2014; Van der Walt and De Klerk, 2015). Self-transcendence is an integral element of meaning and spiritual well-being (Worth and Smith, 2021). Through self-transcendence, individuals shift from being inwardly focused to connecting with something greater than themselves (Frankl, 1984) and contributing value to society (Wong, 2016). Spiritual well-being is enhanced by meaningful relationships with friends, family, nature and the universe, and a sense of belonging and connectedness with the broader community (Miller and Foster, 2010). Spiritual wellness enables overcoming adversity, promoting resilience and life satisfaction, and guarding against burnout (Shelton et al., 2020), enabling executives to move beyond their circumstances towards a higher purpose (Gavin et al., 2003).

### 2.3. The senior executive role and anti-meaning

Research points to several aspects that almost unavoidably accompany the work role of being a senior executive, potentially affecting their well-being adversely (Table 2).

**TABLE 2:** Adverse workplace impacts experienced by senior executives.

Adverse impacts	References
Constant pressure; high stress levels; stigma associated with acknowledging stress	Rook et al., 2016, 2019; Harms et al., 2017
Severe time constraints and lack of work-life as balance	Quick et al., 2014; Bloom, 2016; Rook et al., 2019; Rath and Satpathy, 2020
Exhaustion and burnout	Quick et al., 2014; Sirén et al., 2018
Loneliness, isolation, and social exclusion	Quick et al., 2014; Subhashini and Yadla, 2018; Siqueira et al., 2019; Zumaeta, 2019; Van Tilburg, 2021

With a tendency of executives to measure high on work centrality and job involvement (Judge et al., 1995), have low levels of self-awareness (Quick et al., 2014) and to neglect their emotional well-being (Sirén et al., 2018; Jones, 2019), the adverse workplace impacts on executives (Table 2) are often exacerbated. Experiencing loneliness and isolation places them at risk of social disconnection and unhealthy and self-defeating behaviors (Gavin et al., 2003; Quick et al., 2014).

Meaning has conventionally been viewed as a monopolar construct (Nyholm and Campbell, 2022), albeit on a continuum ranging from meaningful to meaningless. A meaningless life suggests one that is devoid of meaning (Nyholm and Campbell, 2022) and without a sense of purpose or direction (Steger, 2018; Salicru, 2021). It has been widely demonstrated that meaninglessness has a negative impact on well-being and is associated with a range of psychopathologies (Steger, 2018; Vanhooren, 2019; King and Hicks, 2021; Salicru, 2021). Several scholars have suggested that meaninglessness is not the antithesis of meaningfulness in that the absence of something (e.g., meaninglessness) does not equate to its opposite (e.g., meaning) (Metz, 2012, 2013; Campbell and Nyholm, 2015; Nyholm, 2021; Nyholm and Campbell, 2022). Rather, they argued that meaningfulness should be viewed as being a bipolar construct in that its opposite is a construct called anti-meaning. The discourse on anti-meaning originated from accumulating concerns regarding the blemishes and burdens imposed involuntarily on individuals, mainly as consequences from their economic and lifestyle choices (Campbell and Nyholm, 2015). Anti-meaning was defined a as work–life force that directly opposes meaning; something negative that reduces the net amount of meaning an individual experiences (Nyholm and Campbell, 2022). Anti-meaning originates from one’s subjective negative feelings, emotions, and judgments that affect one’s life and activities and impede one’s sense of meaning (Campbell and Nyholm, 2015; Nyholm and Campbell, 2022). For example, excessive work centrality and job involvement in executives often result in putting in extra hours at the office (Judge et al., 1995) to gain a promotion may result in less time with one’s spouse and cause marital strain. The resultant anti-meaning can both diminish the meaning of the promotion and damage the interpersonal relationship, eliminating the experience of meaningfulness from the achievement. The adverse consequences related to the role of a senior executive (Table 2) indicate several potential sources of anti-meaning, which are likely to distract from executives’ net experience of meaning and overall well-being.

Given the significant influence of meaning on well-being (De Jong et al., 2020; Salicru, 2021; Arslan and Allen, 2022), the experience of meaning is crucial to executives’ pathway to attain well-being. However, there is little research available that specifically focuses on senior executives’ experience of meaning. It is however apparent that unavoidable anti-meaning tends to accompany the role of a senior or top executive and is likely to generate additional anti-meaning. The monopolar view of meaning distinctly fails to account adequately for executives’ lived experiences in which anti-meaning is likely to subtract from their overall sense of meaning. Yet, empirical research regarding anti-meaning – still a novel concept – is negligible (Nyholm and Campbell, 2022), specifically as it relates to senior executives’ experiences. There is a discernable need for deeper insight into how meaning and anti-meaning as a bipolar concept influences senior executives’ net experience of meaning in life and well-being. The aim of this study was thus focused on exploring a pathway to greater meaning in life and well-being for senior executives beset by anti-meaning.

## 3. Research method

### 3.1. Study design and sampling

The research represented a phenomenological approach, adopting “a constructivist/interpretivist epistemology, to glimpse into the lived experience of individuals” and their perceived experiences of their situational reality (Dwyer and Emerald, 2017, 5). We utilized a narrative inquiry (NI) study design to elicit rich data (Bruce et al., 2016; Dwyer and Emerald, 2017), by exploring how participants’ respective experiences created their stories (Tamminen and Bennett, 2017). The NI design was chosen since the meaning-related concepts explored are often abstract in nature, personalized and contextual (Steger, 2019). The narratives were embodied by storytelling, facilitated through semi-structured interviews during which participants shared their stories of being a senior executive. The inquiry focused on the participants’ lived experiences, to illuminate their encounters of meaning and the potential factors contributing to anti-meaning in a systematic, but rich way (Bruce et al., 2016). Semi-structured interviews were selected to gather data rather than in-depth interviews to assist with theoretical *a priori* consistency (LaDonna et al., 2021) of the data with conceptualizations of meaning and anti-meaning as bipolar constructs (Campbell and Nyholm, 2015; Nyholm and Campbell, 2022). Ethical clearance (No. 25555) for the research was obtained from the Stellenbosch University Research Ethics Committee.

Available contact details were used to invite chief executive officers (CEOs) and managing directors (MDs) in southern and eastern Africa to participate in the study, followed by snowball sampling, which entailed asking CEOs and MDs to identify other potential participants. Due to the personal nature of the research, several participants opted to approach potential participants themselves in the snowball sampling process, rather than providing contact details to the researchers. To ensure the trustworthiness of data and findings, participants needed to be in the role of CEO or MD for at least five years, as the primary inclusion criterion. Table 3 provides information regarding the sample and participants’ respective contexts. To ensure the anonymity of participants and the confidentiality of contributions, participants’ names were replaced with codes (P1 – P8).

**TABLE 3:** Sample description.

Participant	Age	Position & industry	Years of CEO/MD experience	Marital status	Children
P1	40–50	CEO (Pharmaceutical industry)	14	Married	2
P2	20–30	CEO/business owner (Crypto industry)	10	Married	Expecting
P3	50–60	MD/business owner (Financial services)	20	2nd marriage	2 biological 2 non-biological
P4	30–40	MD (Healthcare services)	10	Married	1
P5	40–50	CEO/business owner (Crypto industry)	5	2nd marriage	0
P6	30–40	MD/business owner (Financial services)	10	Divorced, cohabitating	2 non-biological
P7	60–70	MD/business owner (Multiple industries)	38	2nd marriage	2 biological 2 non-biological
P8	60–70	MD/business owner (Multiple industries)	33	Married	3

Eight male participants were included in the final sample. The aim was to include an equal proportion of male and female participants. Although several female senior executives were directly approached by the researchers and several more were approached by participants in the snowball sampling process, but none followed through with scheduling an interview. Similarly, we approached more than 10 employed CEO/MDs to participate in the research, and several more were approached by participants through snowball sampling. However, few responded favorably and as a result, the sample was skewed towards owner-CEOs.

### 3.2. Data collection and analysis

An interview guide, containing twelve introspective open-ended questions was compiled *a priori* from related theories discussed in the literature study (LaDonna et al., 2021) to engage with participants regarding their experiences. Four of the questions explored participants’ experience of meaning, three questions related to their experiences of anti-meaning (phrased as negative experiences, factors or feelings), two questions related to the relationship between the CEO role with meaning and anti-meaning, and one question related to value–enactment alignment. The relationship between meaning in life and well-being has been so well established (Park and Baumeister, 2017; Pattakos and Dundon, 2017; Steger, 2018; De Jong et al., 2020; Shelton et al., 2020; Li et al., 2021; Salicru, 2021; Arslan and Allen, 2022) that it hardly needed to be confirmed empirically. Notwithstanding, the interview guide included one question that directly explored participants’ well-being. In addition, some of the other questions and follow-up questions touched on how executives’ experiences of meaning and anti-meaning related to their well-being. The relationship between meaning and well-being was further assumed from the vast existing body of knowledge. The last question invited participants to share more observations relating to their lived experiences of well-being, meaning and aspects that negatively influence meaning (i.e., anti-meaning). To promote the generation of rich data, the interviews were conducted in a conversational manner, albeit focused on the research topic through adherence to the interview guide, enriched by thoughtful follow-up questions resulting from participants’ in-the-moment responses (LaDonna et al., 2021).

The researcher who conducted the interviews was a practicing psychiatrist, satisfying the necessity for competent interviewing skills (LaDonna et al., 2021). Although the researcher’s profession was never communicated, her interviewing competence were likely to augment participants’ perception that they were in “trustworthy hands” (Andrews, 2021, 363), enhancing the credibility of the interviews and the trustworthiness of the data and findings (Stenfors et al., 2020). Six interviews were conducted via Zoom or Microsoft Teams and two were conducted face-to-face, as per participants’ preferences. Despite the relatively small sample size, data sufficiency and saturation (LaDonna et al., 2021) emerged from Interview 6, evidenced by no new recurring thematic patterns coming to the fore (Leese et al., 2021), demonstrating sufficient information power for the comprehensiveness of this sample (Malterud et al., 2016). It was the case in this study, LaDonna et al. (2021) noted that data sufficiency can be achieved from a relatively small sample, in a focused study with targeted participants, using theory *a priori* to describe a phenomenon through focused questions.

All interviews were recorded and transcribed verbatim by the researchers for the purpose of analysis. Trustworthiness of the analysis and findings was enhanced by following a systematic approach in conducting the study (Saldaña, 2011). A thematic analysis of the data was done by the first researcher using ATLAS.ti software, based on the six-phase recursive process described by Clarke and Braun (2013). Although knowledge generated through social research can never be entirely objective (Dwyer and Emerald, 2017), aligning the interview questions and data analysis with theoretical conceptualizations, supported by evidence from participants’ stories, enhanced the objectivity and trustworthiness of the NI (Andrews, 2021). As a final step to enhance trustworthiness, the second researcher independently reviewed the coding process and analyses, identifying and addressing apparent inconsistencies and suggesting applicable amendments until a consensus was reached.

## 4. Research findings

The key themes and subthemes from the thematic analysis are noted in Table 4.

**TABLE 4:** Key themes and subthemes.

Key themes	Subthemes
Senior executives find meaning in their work role and relationships.	The senior executive role is a primary source of meaning. Executives can find meaning in interpersonal relationships.
The senior executive role generates unavoidable anti-meaning.	Time demands and constraints generate unavoidable anti-meaning. High stress generates unavoidable anti-meaning. Loneliness is a source of anti-meaning. The image of senior executives promotes anti-meaning.
Anti-meaning can be tempered or aggravated.	Meaning through multidimensional sources tempers anti-meaning. Neglect of wellness generates additional anti-meaning.
The executive role obstructs anti-meaning tempering.	Excessive demands obstruct healthy coping mechanisms, rendering anti-meaning tempering problematic. Lack of systemic support obstructs anti-meaning tempering.

The themes and sub-themes are discussed in the following sections. Although the themes and subthemes represent findings in a useful and compartmentalized manner, they remain interdependent. Some overlap is therefore unavoidable in the discussions. Limited representative excerpts from the interviews are presented verbatim as evidential contributions to the discussion.

### 4.1. Theme 1: senior executives find meaning in their work role and relationships

#### 4.1.1. Subtheme 1.1: the senior executive role is a primary source of meaning

Participants found the role of a senior executive to be highly satisfying and a primary source of meaning. Those who were business owners saw their businesses as an extension of themselves. Participants generally indicated that they derived some sense of meaning and purpose through the potential positive impact that they could have on others – be it assisting people in need or improving people’s lives and circumstances.

*I have the chance, privilege, and grace, to be doing, to be living my purpose. Always in the business of work–lifecreating things, especially things that didn’t exist before*… *which is something that can potentially change the world going forward.* (P2)

This finding confirms the significant role work can play in an individual’s experience of meaning (De Klerk, 2023), and the important role of self-transcendence in finding meaning (Pattakos and Dundon, 2017). Most participants regarded their position as something meaningful to which they had aspired and felt that they had reached a personal goal.

*If I had to do it all over again, I probably would have done it exactly the same*… *it is still what excites me*… *It’s who I am. (P6)*

*That’s what I’ve worked for, to be in this position, so that I can do what I wanted to do. It’s at my core.* (P7)

All participants experienced a sense of meaning from being a provider for their families, employees (and their families), shareholders, and broader society, and from providing security and creating opportunities. Achieving their business goals provided participants with a broader sense of purpose.

*That’s the role and purpose in life – to be the provider and protector of my family and my team and my staff.* (P5)

This finding confirms that individuals can derive a sense of purpose and meaning through attaining personal goals (Martela and Steger, 2016). It was not only reaching financial goals that drove participants’ actions, but also self-transcendence through the ability to impact others positively.

*I’m comfortable that I’ve done something which has made somebody else’s life better than it was.* (P3)

For several of the participants, part of the meaningfulness of their position was the fact that it gave them a sense of having autonomy and freedom of choice, even the choice to pay more attention to business demands than to other life domains. Their degree of autonomy was often the justification for business becoming the top priority in their lives – irrespective of any negative effects it could potentially generate.

*Every late night of work, I feel like I’m doing something good and meaningful. I am trying to look after people’s concerns, or sort out something someone wanted me to do, or grow the business. But at the same time, I would rather want to be doing something more fun and I know I am in overtime, and I should actually be sleeping or spending time with my wife, or going to the gym class, or doing sports, or living my life in some way. But I often feel like that’s the price to pay. And it is, it is our choice to do that.* (P5)

Executives’ intrinsically worthwhile role function influences their sense of meaning in life positively, even when associated with aspects that decrease life satisfaction. Participants’ characteristics and values, such as achievement, competitiveness, ambition, and being goal-driven and hard-working, align closely with their senior executive role.

*I always did everything, whether it was sports, I did a lot of sport*…, *but nobody was going to out-train me. I was going to train harder than anybody. And on the business, nobody was going to out-work me.* (P8)

Executives’ ability to self-express and embrace their core values via their work role can distinctly make their work role meaningful (Martela and Steger, 2016) and affirm their identity and self-esteem (Miller and Foster, 2010).

#### 4.1.2. Subtheme 2.1: executives can find meaning in interpersonal relationships

Participants all expressed the overall importance of family (primarily) and friends in their lives. Being a good husband and father was uniformly regarded as how they wanted to be remembered. They considered spending time with family and friends to be a healthy and meaningful way to relax, have fun, and connect.

*I just want to be remembered as a good father and husband. That’s all.* (P4)

*Socializing with people*… *happy moments, laughter, sharing a meal, doesn’t have to be good food, just kind of eating together. It is just about who you are with and enjoy having a laugh together.* (P5)

The top executives evidently found a sense of meaning through rewarding personal relationships – primarily with their families. However, while there was an acknowledgment of the importance of personal relationships, being a good father or husband was usually linked back to the ability to be a good provider. Moreover, while fostering family relationships required spending time with family members, fulfilling the role of provider required time away from family and could thus become a source of anti-meaning.

Most participants experienced marital strain as the demands, pressures, and stresses associated with their work spilled over into their home lives. Several described experiencing a constant energy drain both at work and at home. At work they would be under pressure, stressed, and confronted with complex and high-risk decisions. When they then entered their home space with low reserves, they were often faced with additional problems to which they responded poorly, eliciting negative emotions within themselves or their spouse.

*Having a bad day, having a difficult day, having a stressful day, and coming home to the same problem putting on a different pair of glasses. But I am already drained and it kind of just exaggerates the whole problem. Now the flat tire is not a flat tire anymore. It’s an irritating thing I need to deal with now.* (P6)

This finding confirms the impact of extensive demands on executives’ emotional and psychological wellness (Quick et al., 2014), creating a platform for marital problems. Ostensibly, although interpersonal relationships can be a source of meaning, additional stress and frustration in executives’ marital relationships can become sources of anti-meaning.

### 4.2. Theme 2: the senior executive role generates unavoidable anti-meaning

#### 4.2.1. Subtheme 2.1: time demands and constraints generate unavoidable anti-meaning

The strong sense of meaning derived from the executive role and participants’ identification with it set the stage for the prioritization of work, and thus more time is dedicated thereto than to other life domains. All participants described their executive role as requiring much time and energy. They experienced their lives as being rushed and busy and found it difficult to find time to attend to everything else and everyone that required their attention.

*The availability of time is the biggest challenge [for a senior executive], more than anything else. And then, I suppose, patience and being able to live in a relationship where time is short, and frustrations are high.* (P3)

*Very often my negative emotions are related to my wife*… *I need to try to compensate to keep her happy despite working all the time and that is exhausting and sometimes irritating.* (P5)

Onerous workloads, long working hours, and time pressures contributed to participants’ work-related stressors and personal life stressors. There tends to be a mismatch between the amount of time and attention executives have available and what is demanded of them. Time pressure was not only an unavoidable source of anti-meaning that elicited negative feelings, but it also generated additional anti-meanings such as resentment.

*If I’m supposed to be back home at a particular time and I’m late, or if I’m not responding on something or engaging on something from the business side, the negative response could be a feeling of resentment because I’m neglecting or perceived to neglect a particular concern of others.* (P5)

Long work hours affected participants’ ability to attain a sense of well-being. Participants were often left feeling inadequate in their ability to find or maintain a healthy balance between work and life and to dedicate their non-work time effectively across other life domains such as family, leisure and exercise.

*My position, my job, is a, it’s a source of a lot of the difficulties that that I face – from a time perspective, from a relationship perspective, from a spiritual perspective, from a social and recreational perspective. It’s the side of my life which stops me from doing any of those things. So, I think it does impact every aspect of life.* (P3)

*If something important comes up workwise, obviously, I’ll cancel the golf. Then I’d probably feel frustrated, and I’d probably start saying, you know, I’ve got to get this balance right.* (P3)

Digital connectivity has added complexity, as the urgency to respond to and meet certain work-related demands 24/7 has been heightened.

*Technology has blurred the lines between responsibility boxes and time boxes*… *when you can WhatsApp me, live, on a Sunday morning, you’ve knocked me out of my chill zone with something that I need to do for you and I’m in my family time.* (P5)

Substantive workloads and long work hours render time constraints a primary source of unavoidable anti-meaning. The resulting negative consequences of these constraints are especially prominent in executives’ interpersonal relationships and in their ability to achieve a holistic sense of well-being. These findings confirm time constraints as a significant difficulty that senior executives face, resulting in an ever-elusive work–life balance (Quick et al., 2014; Bloom, 2016; Rook et al., 2019; Gragnano et al., 2020), and accentuating the inappropriateness of applying the traditional notion of a work–life balance to senior executives as a pathway to well-being.

#### 4.2.2. Subtheme 2.2: high stress generates unavoidable anti-meaning

Most participants experienced high degrees of responsibility, complex decision-making, and challenging problem-solving expectations, which contributed to high levels of stress, with a negative impact on mental and physical well-being. Because top executives face a unique set of conditions tied to their role, they must function well despite high degrees of stress, thus setting the stage for unavoidable anti-meaning.

… *being under enormous pressure and dealing with high-risk complex decisions and being exhausted by the time you get home.* (P1)
*Workload, lack of sleep, too many decisions with interconnecting dependencies, feeling like everything relied on me, and not being able to achieve everything with the maximum amount of time I could allocate to everything.* (P5)

Dealing with these difficulties, most participants noted that they would sometimes engage in negative and unhealthy, self-defeating behaviors (outbursts, drinking, avoidance, extra-marital affairs, etc.).

*[Executives] drinking, cheating, weekends away, shouting at their loved ones, drinking with their friends, obviously shying away from personal responsibilities.* (P6)

Operating in a high-pressure environment is obviously part of the senior executives’ reality and they cannot escape potential stress-generating factors (Rook et al., 2016, 2019; Harms et al., 2017), which adds to unavoidable anti-meaning. Unhealthy coping mechanisms, therefore, generate further additional anti-meaning.

#### 4.2.3. Subtheme 2.3: loneliness is a source of anti-meaning

Several participants experienced a sense of loneliness that was directly linked to their role as a senior executive. Top executives carry a huge workload and shoulder the highest degree of responsibility and accountability in their organizations. They are also responsible for keeping employees motivated and assisting them with personal problems. Close interpersonal relationships with employees can be awkward as these would skew objectivity when required to address poor performance, bad behavior, or even staff layoffs.

*I cannot just talk to anyone, because sometimes the problem is confidential, due to the nature of the process*… *you do not want to build too many relationships with people working below you, because you need to sometimes make the tough decisions. You cannot just be too close as it influences your judgment when it comes to making the tough decision or leave you with more guilt and stress about how you impacted them negatively.* (P1)

Participants expressed a desire to be able to reach out to someone outside their personal circle to whom they could talk, but regarded discussing their business or personal issues with those who worked under them as inappropriate.

*I sit in the office with everybody and feel completely lonely, at times*… *you’re not part of the team. you kind of feel like an outsider.* (P6)

*And it’s hard because you really often don’t have anyone to talk to because there’s no one you can trust*…, *especially if the information could have a detrimental impact on your business or staff or something. So, you really have to be careful about who you talk to and you trust fewer and fewer people. So, you do get isolated and lonely.* (P5)

These findings confirmed the socioemotional cost of being a high-ranking corporate leader, leading to experiencing loneliness, increased pressure, lack of social support, increased social distance, and exhaustion. Participants felt their spouses and close social circles were more supportive when they had some understanding of their work-related demands and pressures. Participants however also expressed a reluctance to discuss their work-related difficulties with close family and friends. They noted that people in their personal circle often did not understand their problems and they did not want to add additional burdens on them.

*You want to keep the personal relationship without burdening them with your work-related issues. But you also want, after a very busy day*… *you want to spend a happy time with the people that you love. And it’s frustrating for you if they do not understand your problems, or, for them, if you fill the little time you have with them with more business-related stuff.* (P1)

Despite needing an outlet to talk about their problems, the feeling of not being understood by those in their inner circle, especially in relation to a spouse, escalated participants’ reluctance to discuss issues, which increased levels of frustration.

*It breeds negative emotions from your spouse. And then it leaves me reluctant to discuss anything. And I am left with nowhere to vent about my work stuff, because who else am I supposed to talk to?* (P6)

These aspects reinforce the experience of loneliness, both at the workplace and at home. When loneliness is experienced both at work and at home, it decreases the ability to temper against workplace loneliness and can result in an array of additional negative outcomes (Quick et al., 2014; Subhashini and Yadla, 2018).

#### 4.2.4. Subtheme 2.4: the image of senior executive promotes anti-meaning

Participants felt as if they operated in an environment that is competitive and unforgiving. They experienced a requirement to function effectively in this environment, growing the business, increasing profits, retaining the trust of employees and shareholders, and outperforming competitors – thus being a kind of superhuman.

*Senior executives are normally expected to be the superheroes – always strong, rock-solid decisions are made, sharp, good negotiation skills, etc. All of this requires, like it has an image, that they are superheroes in that domain. And many times, they are, at the end of the day, they are human beings.* (P1)

Participants felt a need to portray an outward façade of being strong and in control, irrespective of their emotional experiences of stress and exhaustion. Nevertheless, most participants identified with the projection of being superior, reinforcing the notion that they were not like the average person, did not have the same difficulties as the average person, and did not require the same help as the average person.

*I used to think I was really, really, I think amazing. Until I had this brain burnout last year. Before then, I thought I could do anything and that I didn’t really need to sleep. And would think all these rules that people talk about don’t apply to me.* (P5)

Given the importance of consistently trying to portray a superhuman image, most participants felt that reaching out to ask for help would be seen as a sign of weakness.

*You can’t show any weakness*… *and some of these people don’t even acknowledge what they’re doing or are too afraid of being branded as being weak.* (P6)

*A CEO of a big, big, big business*…, *he’s not going to come and tell me I’m stressed or unhappy*… *because he wouldn’t*… *want to show weakness.* (P7)

This finding confirms the stigmatization and reluctance of top executives to openly talk about their emotional problems or to reach out for help when needed (Quick et al., 2014; Rook et al., 2016, 2019; Harms et al., 2017; Jones, 2019). This view of themselves raised the issue of where to find help that is credible, appropriate, and effective.

*Something that I would like*,… *somebody that I can go talk to that’s also an intellectual, that understands organizational structure, but understands mental problems, or challenges, for that matter.* (P6)

*It needs to be something credible from someone you respect.* (P2)

Participants’ estimation of their capabilities is not only a barrier to acknowledging a perceived weakness, but also creates a credibility barrier a helping party to be equally as capable and credible as they perceive themselves to be.

### 4.3. Theme 3: anti-meaning can be tempered or aggravated

#### 4.3.1. Subtheme 3.1: meaning through multidimensional sources tempers anti-meaning

Apart from noting the importance of significant work as a source of meaning in life, two (older) participants emphasized the important contribution of other life domains to achieving such meaning, for instance self-transcendence, interpersonal relationships, and spirituality (contribution to the greater good outside business-related efforts).

*I would certainly be living a very meaningful life and a very purposeful life. I’ve always been on countless boards and committees and countless social endeavors. So, yes, I think overall I actually have made a meaningful contribution in life to myself and society*… *I like to think of myself as being somebody who’s always there to help other people when I can.* (P8)

By sourcing meaning multidimensionally, executives became less inclined to find meaning only via their work role and they felt more content with the degree of meaning they experienced in their lives. However, this was apparently easier for older participants who had met their life goals and were now left feeling content.

*I’m very happy. I think I’ve got a nice balance in terms of friends and relationships. And I think I’ll do my best to contribute to the broader well-being of society and community.* (P7)

*Now I’m at the end of my career, I guess spending time with friends and family and recreation are the things that, really, I enjoy the most. I feel mission accomplished. I feel that I’ve done what I set out to do.* (P8)

Generating meaning from life domains other than work seemed to have had a tempering effect against anti-meaning. In contrast, when work became central to participants’ sense of meaning, prioritizing this role often came at the expense of other life and wellness domains. Executives’ dedication to their work demands can thus limit their ability to dedicate time to other personally valued life domains and activities, and participants generally acknowledged the impact thereof on their experience of meaning.

*I would probably say 50/50 [extent of living a life of meaning], for instance, being in the medical field, I’m able to help a lot of people. But I’m not getting to do stuff that I want to do. I think that’s probably more the frustrating part of this. You can always do more on your own personal space, but work keeps you busy.* (P4)

It is evident that, although the executive role can generate meaning, it can also negatively impact executives’ ability to source meaning via other important life domains – generating potential anti-meaning, which directly deducts meaning from the initial activity.

#### 4.3.2. Subtheme 3.2: neglect of wellness generates additional anti-meaning

Several participants acknowledged that maintaining good physical health and good social connections can be a challenge. Most participants found sports and exercise to be good outlets for their stress and frustration and to be a central part in keeping them physically and mentally agile and able to continue to function effectively in a high-pressure environment.

*I worked really hard. And I did do individual sports because I needed that outlet.* (P8)

*I realized I work a lot of hours, like an insane number of hours, in general. And I realized if I do not exercise and sit at a desk all day long, then it gives me a compounded effect. So, then my psychological well-being issues will be bigger, my stress will be higher. So, it just gives me a release.* (P1)

The emphasis on the role of sport and exercise contributed to participants’ ability to temper the stress from their high-pressure work, schedules, and responsibilities. Exercise and participation in sports can be an important means of releasing stress and promoting well-being (Quick et al., 2014) and a healthy means of tempering against unavoidable anti-meaning. However, most participants struggled to maintain physical wellness over a sustained period.

*I’m working too much. I’m not doing enough sports. I have to start working out again. And I’m eating really bad. And then socially*… *I’m very lucky to have real genuine friends. I don’t meet them too often now, because I’m quite busy and, also, don’t make a lot of effort.* (P2)

*Physical activity is important*… *[Executives] don’t have the time to exercise, for example, or eat healthily because you’re just on the run all the time, and also because our positions require a lot of travel. This means what comes with it: lack of sleep, drinking, eating late.* (P1)

This finding confirms that, despite senior executives requiring physical well-being to allow them to physically keep up with their job demands (Quick et al., 2014), time constraints often hinder long-term attention to physical wellness.

Participants placed great value and meaning on interpersonal relationships, but this domain was often negatively affected by their work role. Participants’ dedication to social wellness – through relationships and spending time with their spouse, children, family and friends – was frequently impeded by lack of time availability. With participants focusing on addressing their vocational demands, time constraints often resulted in other wellness domains being neglected. Participants emphasized how they faced difficulties in maintaining healthy relationships, despite such relationships being greatly valued.

*My daughter started Grade 1 now. So, it’s starting to become a bit more difficult because she does sport a lot of time during my working hours. So, it’s becoming increasingly more difficult to balance my time because I’ve got deadlines and things to do.* (P4)

Marital difficulties, extra-marital affairs and potentially also divorce were noted as specific risks that resulted in increasing the experience of loneliness.

*I think my first marriage fell apart because I wasn’t available. That’s the reason that it all fell apart. If you don’t work on it, it’s got no chance. And if you’re not available, it’s got no chance. So, I think it’s important for me to be available.* (P3)

*He [an executive] comes home and his wife is just nagging and just being a pain and sending a lot of negative emotions. And that tends to lead to a lot of these guys cheating as they are already frustrated and feeling alone in what they are facing.* (P2)

While most participants expressed some awareness of a higher power, none of the participants found their life meaning to be related to the domain of spiritual wellness or finding meaning in it. Their higher-order goals were all work-related.

*But it’s a deeper level of connection to something and I don’t even really know what that something is. I think it relates to my sense of loneliness, in the sense that when you connect with this deeper thing, you don’t feel alone. Yeah, I have a purpose. I don’t know.* (P6)

As spirituality has been placed at the center of both meaning (Frankl, 1992) and well-being (Miller and Foster, 2010), participants’ lack of attention to this domain resulted in a loss of a significant potential source of non-work-related meaning, and instead generated anti-meaning by limiting their ability to find transcendental meaning (Wong, 2016).

Most participants noted an awareness of the importance of psychological well-being and rated this well-being as good. However, only two participants indicated that they actively sought ways to improve their stress resilience and coping mechanisms. Several participants revealed experiencing episodes of anxiety, depression, high levels of frustration, irritability, burnout, heightened discomfort, conflict, and an inability to connect with people or properly communicate their own emotions and feelings. Participants made it clear that they were falling short of reaching holistic well-being. They generally granted greater priority to financial and work demands. Most participants indicated limited or no attempts to find assistance to address their psychological well-being issues to enhance their mental wellness.

*Stress management, depression, lying kind of, as you are putting up a facade of what you need to put out. This is reality.* (P5)

*I didn’t manage my anxiety well. I never had medication or anything like that. Didn’t sleep well. I think I just threw myself into activities all the time to keep busy all the time. And by keeping busy, you don’t have to sort of confront the demons.* (P8)

Addressing aspects relating to emotional and psychological well-being can build resilience and stress tolerance (Rook et al., 2016, 2019; Harms et al., 2017; Park and Baumeister, 2017), thereby creating a temper against the unavoidable anti-meaning associated with the senior executive role. Not paying attention to emotional and psychological well-being can, in contrast, weaken resilience to stress and stress tolerance and affect multiple life domains negatively, and thus generate additional anti-meaning. In neglecting psychological, spiritual, and social well-being domains, executives limit their ability to source meaning multidimensionally to form a temper against unavoidable anti-meaning (such as stress and loneliness) and risk generating further additional anti-meaning.

### 4.4. Theme 4: the senior executive role obstructs anti-meaning tempering

#### 4.4.1. Subtheme 4.1: excessive demands obstruct healthy coping mechanisms, rendering anti-meaning tempering problematic

Participants often felt inadequate in their ability to find or maintain a healthy balance between work and life and to dedicate their non-work time effectively across other life domains such as family, leisure, and exercise.

*Negativity comes out of getting a bit unbalanced in terms of health and work. And that can be finger pointed at lack of routine exercise, lack of following a meaningful diet, partying it up every night, drinking too much. That’s always negative because one wakes up and thinks, well, that’s not sustainable.* (P7)

Most participants noted the positive impact of having a good support system and the need to find the time and space where they did not have to think about work.

#### 4.4.2. Subtheme 4.2: lack of systemic support obstructs anti-meaning tempering

All participants noted the general lack, but importance and value, of having support systems in place for senior executives. Well-being programs and support systems that were developed for the benefit other employees did not appropriately deal with the specific needs and issues of these executives; did not adequately address issues regarding the specific expectations and potential stigma they faced; and were not regarded as credible by the executives themselves.

*The senior level people, I think, should go away for a week or two – a mental health farm – where they can relax. And I think that should be mandatory for senior-level staff*… *companies don’t really do much in this avenue, no matter what they say.* (P7)

The organizations of all the participants offered some form of mental wellness support program targeting general employees. However, participants felt that senior executives were either overlooked, not expected to attend, or that these programs did not cater to the needs of senior executives.

*We also need a bit of support*… *just a person who phones in and has a discussion with you, to check in on how things are going with you or if there is anything that they can help you with so that you don’t feel that everything is on you. So, it would be supported in a wellness space.* (P4)

This observation illustrates the need for raising awareness among, and building credibility with, senior executives. There appears to be a lack of deeper awareness and broad incorporation of mental wellness needs for those individuals at the top of the hierarchy. This lack of systemic support is likely to promote unavoidable anti-meaning tied to the senior executive function (such as time constraints, stress, and loneliness).

## 5. Discussion

**FIGURE 1:**
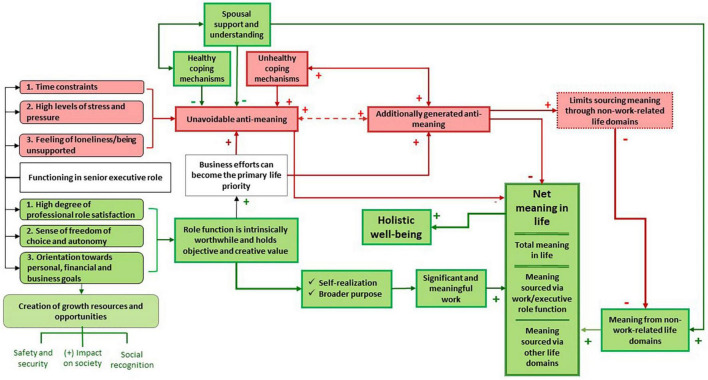
Meaning and anti-meaning experienced by senior executives.

We demonstrated how the role of senior executive provides individuals with a significant sense of meaning (Martela and Steger, 2016). However, this role tends to create unavoidable anti-meaning for executives, whereafter additional anti-meaning is generated which further diminishes executives’ overall sense of meaning (Campbell and Nyholm, 2015; Nyholm and Campbell, 2022). At best, these occurrences of anti-meaning can hinder senior executives from experiencing meaning in life to its fullest extent. At worst, they can neutralize the experience of meaning. In contrast, when meaning takes on a multidimensional scope, it creates a buffer against anti-meaning by making senior executives’ sense of meaning less dependent on their professional success (Campbell and Nyholm, 2015; Nyholm and Campbell, 2022). Although executives who source meaning multidimensionally are still faced with certain levels of unavoidable anti-meaning, they can better create a buffer against anti-meaning and limit its overall negative impact (Gavin et al., 2003; Rosso et al., 2010; Wong, 2016). Figure 1 was derived from the research findings to demonstrate the systemic interrelatedness between some of the most important aspects involved in executives’ experiences of meaning, anti-meaning and anti-meaning tempers, in relation to their well-being.

In Figure 1, we depict how the senior executive role is associated with both positive and negative elements in relation to meaning. The role provides executives with components that make their work intrinsically meaningful (green blocks). Ashmos and Duchon (2000), Pradhan and Jena (2016). However, senior executives’ work sometimes becomes so dominantly meaningful that it can dictate their lives (Quick et al., 2014), to the extent that it generates unavoidable anti-meaning (red blocks) (in the form of time constraints, high levels of work-related stress and pressure, loneliness, etc.) (Quick et al., 2014; Rook et al., 2016, 2019; Harms et al., 2017; Subhashini and Yadla, 2018). If insufficiently tempered, unavoidable anti-meaning may act as the catalyst for additionally generated anti-meaning (red blocks) (Campbell and Nyholm, 2015; Nyholm and Campbell, 2022). Once additional anti-meaning has been generated, the presence of either unavoidable or additional anti-meaning can reinforce the generation of the other. Top executives can temper against the unavoidable anti-meaning through healthy coping mechanisms and spousal support and understanding.

**FIGURE 2:**
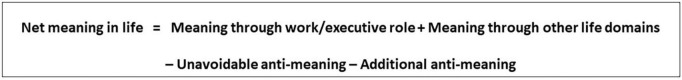
Net meaning in life equation.

However, unhealthy coping mechanisms tend to enhance both the amount of unavoidable anti-meaning and generate additional anti-meaning, which can deduct from the total amount of meaning experienced and limit executives’ ability to source meaning via other life domains (Park and Baumeister, 2017), thereby generating additional anti-meaning. An executive’s total experience of meaning can thus be approximated by adding together the meaning sourced via their work and meaning sourced via other life domains. From this total, unavoidable anti-meaning and additionally generated anti-meaning need to be deducted to reflect the net experience of meaning. From this discussion, the equation for estimating net meaning in life was derived (Figure 2).

### 5.1. Practitioner applications

**FIGURE 3:**
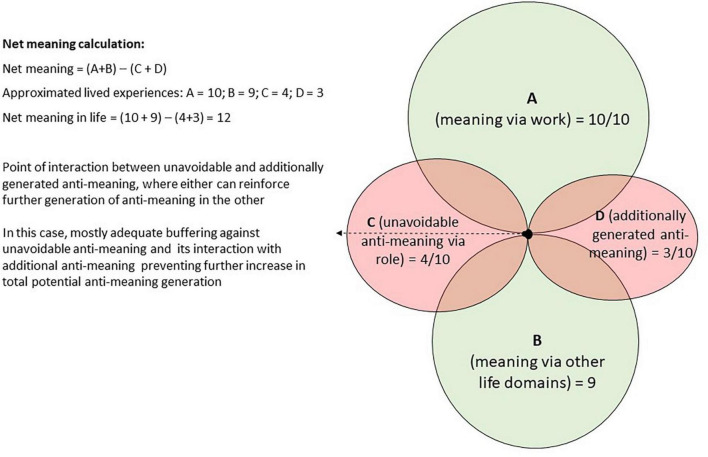
Graphical representation of a hypothetical executive’s lived experience.

The net meaning in life equation (Figure 2) provides practitioners with an effective pathway to identify important areas of focus when counseling senior executives, especially when administering the pathway graphically (Figure 3). The graphical presentation consists of four overlapping circles, representing different sources of meaning (via work and other domains) and potential anti-meaning (unavoidable and additionally generated) that could be experienced by any senior executive. The circles represent the relative sizes of each component. The first step involves careful consideration of components that enhance the executive’s experience of meaning, and those components that represent anti-meaning in the form of negative side-effects. The second step involves allocating hypothetical, but informed, estimated amounts of meaning between 1 and 10, reflecting the approximate degree of meaning and anti-meaning to each component. Rating one’s experience on such a scale is a common approach to elicit a quantified measure of experiences ranging from pain or health (Hirsch et al., 2020) to happiness (Tsutsui et al., 2017). We suggest that quantification of the experiences should be derived through intensive conversations with the executive and their life experiences. The net amount of anti-meaning is then deducted from the net amount of meaning to represent an approximation of the total amount of meaning (Figure 2).

Figure 3 illustrates the hypothetical lived experience ratings of a CEO who finds the executive role meaningful, but also derives some meaning from other life domains, especially family relationships. Although this CEO struggles with unavoidable time constraints and work stress that influence their relationship with their spouse, the CEO sometimes uses healthy coping mechanisms to deal with stress, which creates a buffer against additional anti-meaning.

The net sense of meaning for the executive in Figure 3 can be estimated as (A + B)–(C + D) (Figure 2). Thus, the net life meaning of this CEO is approximated to be 12/20 (60% meaningfulness). This scenario illustrates the importance of sourcing meaning multidimensionally for an executive’s ability to experience meaning, despite the presence of unavoidable anti-meaning and additionally generated anti-meaning. In this case, practitioners could encourage the CEO to source meaning multidimensionally, focus on limiting current sources of unavoidable anti-meaning and strengthening anti-meaning buffers to optimize the executive’s overall sense of meaning.

**FIGURE 4:**
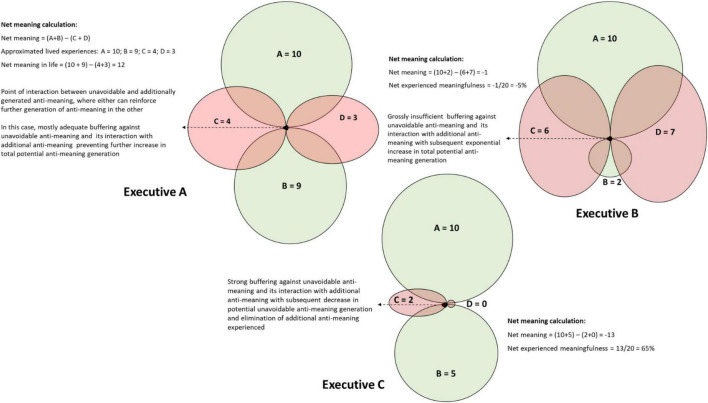
Graphical representation of different hypothetical executives’ lived experiences.

Similar to the scenario presented in Figure 3, this graphical pathway can be applied to other hypothetical illustrative cases as indicated in Figure 4.

In Figure 4, Executive A needs guidance on finding sources of meaning also outside the workplace. More importantly, this executive needs assistance on how to develop mechanisms to alleviate unavoidable anti-meaning and ways to create buffers against additional anti-meaning. This scenario accentuates the importance of tempering against the unavoidable anti-meaning faced by senior executives. When senior executives are not able to erect buffers against unavoidable anti-meaning, it not only enhances the negative impact of the unavoidable anti-meaning itself, but also stimulates the further generation of additional anti-meaning. With a negative meaning score, Executive B is on the cusp of existential burnout due to excessive anti-meaning. This scenario accentuates the potential issues stemming from relying solely on work for a sense of meaning; an inability to temper the unavoidable anti-meaning tied to the senior executive role; and not employing healthy coping mechanisms to create a buffer against additional anti-meaning. When practitioners deal with such cases, they need to provide guidance that will alleviate the negative impact of additionally generated anti-meaning while focusing on measures to limit the impact of unavoidable anti-meaning. Once the immediate threats of anti-meaning have been sufficiently addressed, practitioners can encourage the executive to source meaning more multi-dimensionally. Executive C is on a good track, having the ability to set up buffers to additional anti-meaning in a healthy way but would do well with guidance on how to find meaning outside work. This scenario reinforces the importance to source meaning multidimensionally and to employ a variety of tempering mechanisms against unavoidable anti-meaning.

### 5.2. Limitations and recommendations for future research

Although this study adds to the still limited body of research on meaning in life and the well-being of senior executives, there are some limitations. Although data saturation and sufficiency were observed, the study population consisted of a small sample of only male participants, representing only southern and eastern Africa. We can only speculate how women executives might experience meaning and anti-meaning. However, due to the stereotyping of women in the workplace, it appears plausible that they are likely to be subjected to even more intense anti-meaning than their male counterparts due to role conflict (Chang and Milkman, 2020). A larger population and more representative size would have added to the trustworthiness and generalizability of the findings. However, it appears likely that employed CEOs might experience even more intense anti-meaning than their ‘business owner’ counterparts as their associations with their companies are likely to contain less entrepreneurial ownership (Van der Zwan et al., 2016). Participants represented largely business owners, therefore the study’s findings may be more applicable to owner CEOs than employed CEOs. Nevertheless, as the characteristics of the participants as CEOs are likely to be, at least somewhat similar to other senior executives, notwithstanding gender and regionality, it can be argued that the findings can considered transferable beyond the limitations of this study (Daniel, 2019). As the relationship between meaning in life and well-being has been so well established, the interview guide contained only one question that directly explored well-being (apart from follow-up questions and other questions indirectly relating to well-being), rather than exploring well-being in more detail.

Further studies are required to grow the limited body of research regarding the well-being and experience of meaning by senior executives. Future studies could include larger sample populations and proportional representation between genders, different industries, geographical locations, and participants from a wider variety of industries and types of businesses to improve the generalizability of the findings. Particular attention could be paid to the impact of the senior executive role and the spousal relationship on meaning and anti-meaning. More comprehensive research is needed to determine which form of systemic support would be best suited to address the specific well-being and meaning-in-life needs of senior executives. Although initial applications of the pathway to greater meaning in life and well-being for senior executives beset by anti-meaning (Figures 3, 4) have already yielded promising results in counseling senior executives, this model has not yet been subjected to rigorous research. More research on the application of this model is required to confirm its scientific trustworthiness.

### 5.3. Conclusion

There has been a growing acknowledgment of the importance of well-being among employees, but senior executives, as a group, have largely been overlooked in this respect (Jones, 2019). Viewing meaning in life as a construct with bipolar dimensions can aid in conceptualizing how senior executives experience meaning and in guiding them to reach greater levels of meaning and well-being, notwithstanding their seemingly unattainable ideal work–life balance. In this study, we empirically derived a pathway to improve executives’ sense of meaning, temper unavoidable anti-meaning and to limit the generation of additional anti-meaning. We then demonstrated how practitioners can use this pathway as an effective approach to optimize senior executives’ experience of meaning and thereby improve their well-being. The empirically derived model (Figure 1) and newly derived net life-meaning equation (Figure 2) provide a useful method to assist executives with a life meaning pathway to temper anti-meaning and enhance their well-being. The graphical illustration of meaning and anti-meaning (Figures 3, 4) is likely to resonate with the practical realism of executives, providing them with an effective pathway to greater meaning in life and well-being. Incorporating the construct of anti-meaning in conceptualizing the degree to which senior executives find their lives to be meaningful, provides a promising and practical approach to better support the needs of this important, yet often overlooked group of high-ranking individuals.

## Data availability statement

The raw data supporting the conclusions of this article will be made available by the authors, without undue reservation.

## Ethics statement

The studies involving human participants were reviewed and approved by Stellenbosch University Research Ethics Committee: Social, Behavioural and Education Research (SBER). The patients/participants provided their written informed consent to participate in this study.

## Author contributions

J-MK conceptualized the research and conducted the empirical work and analyses under the supervision of and in collaboration with JD. JD drafted the final version of the manuscript. Both authors contributed to the article and approved the submitted version.
